# The efficacy and safety of bevacizumab beyond first progression in patients treated with first-line mFOLFOX6 followed by second-line FOLFIRI in advanced colorectal cancer: a multicenter, single-arm, phase II trial (CCOG-0801)

**DOI:** 10.1007/s00280-012-1948-1

**Published:** 2012-08-12

**Authors:** Goro Nakayama, Keisuke Uehara, Kiyoshi Ishigure, Hiroyuki Yokoyama, Akiharu Ishiyama, Takehiko Eguchi, Kenji Tsuboi, Norifumi Ohashi, Tsutomu Fujii, Hiroyuki Sugimoto, Masahiko Koike, Michitaka Fujiwara, Yuich Ando, Yasuhiro Kodera

**Affiliations:** 1 Department of Gastroenterological Surgery (Surgery II), Nagoya University Graduate School of Medicine, 65 Tsurumai-cho, Showa-ku, Nagoya, Japan; 2 Department of Surgical Oncology (Surgery I), Nagoya University Graduate School of Medicine, Nagoya, Japan; 3 Department of Surgery, Konan Kosei Hospital, Konan, Japan; 4 Department of Surgery, Komaki City Hospital, Komaki, Japan; 5 Department of Surgery, Okazaki City Hospital, Okazaki, Japan; 6 Department of Surgery, Nakatsugawa City Hospital, Nakatsugawa, Japan; 7 Department of Surgery, Tosei Hospital, Seto, Japan; 8 Department of Clinical Oncology and Chemotherapy, Nagoya University Hospital, Nagoya, Japan

**Keywords:** Colorectal cancer, Chemotherapy, Bevacizumab beyond progression (BBP)

## Abstract

**Purpose:**

The aim of this study was to evaluate the efficacy and safety of the planned continuation of bevacizumab beyond first progression (BBP) in Japanese patients with metastatic colorectal cancer (mCRC).

**Methods:**

Previously untreated patients with assessable disease were treated with mFOLFOX6 plus bevacizumab until tumor progression, followed by FOLFIRI plus bevacizumab. The primary endpoint of the study was the second progression-free survival (2nd PFS), defined as duration from enrollment until progression after the second-line therapy. Secondary endpoints of the study were overall survival (OS), survival beyond first progression (SBP), progression-free survival (PFS), response rate (RR), disease control rate (DCR), and safety.

**Results:**

In the first-line setting, 47 patients treated with mFOLFOX6 plus bevacizumab achieved RR of 61.7 %, DCR of 89.4 %, and median PFS of 13.1 months (95 % CI, 8.7–17.5 months). Thirty-one patients went on to receive a second-line therapy with FOLFIRI plus bevacizumab and achieved RR of 27.6 %, DCR of 62.1 %, and median PFS of 7.3 months (95 % CI, 5.0–9.6 months). Median 2nd PFS was 18.0 months (95 % CI, 13.7–22.3 months). The median OS and SBP were 30.8 months (95 % CI, 27.6–34.0 months) and 19.6 months (95 % CI, 13.5–25.7 months), respectively. No critical events associated with bevacizumab were observed during the second-line therapy.

**Conclusion:**

The planned continuation of bevacizumab during a second-line treatment, BBP strategy, is feasible for the Japanese mCRC patients.

## Introduction

Colorectal cancer is one of the most common cancers worldwide and remains the third leading cause of cancer-related mortality in Japan [1, 2]. For several years, first- and second-line chemotherapy with 5-fluorouracil (5-FU) and folinic acid (FA) in combination with either irinotecan (FOLFIRI) or oxaliplatin (FOLFOX) had been the standard therapy for metastatic colorectal cancer (mCRC) [3, 4]. More recently, these combinations are used together with bevacizumab, a humanized monoclonal antibody that binds to and neutralizes vascular endothelial growth factor (VEGF). Benefits of adding bevacizumab to either the established first-line or second-line chemotherapeutic regimens have been robustly documented in previous clinical trials. Regarding the first-line treatment, Hurwitz et al. reported that addition of bevacizumab to fluorouracil-based combination chemotherapy showed significantly better clinical outcomes as compared with chemotherapy alone (overall survival [OS]: 20.3 vs. 15.6 months [hazard ratio [HR]: 0.66; *P* < 0.001], progression-free survival [PFS]: 10.6 vs. 6.2 months [HR: 0.54; *P* < 0.001], and response rate [RR]: 44.8 vs. 34.8 % [*P* = 0.004]) [5]. Kabbinavar et al. reported that addition of bevacizumab to fluorouracil/leucovorin (FU/LV) improved survival as compared with FU/LV alone (OS: 17.9 vs. 14.6 months [HR: 0.74; *P* = 0.008], PFS: 8.8 vs. 5.6 months [HR: 0.63; *P* < or = 0.0001], RR: 34.1 vs. 24.5 % [*P* = 0.019]) [6]. Furthermore, Saltz et al. reported that addition of bevacizumab to oxaliplatin-based chemotherapy significantly improved PFS, although OS did not reach statistical significance, and the RR was not improved (PFS: 9.4 vs. 8.0 months [HR: 0.83; *P* = 0.0023], OS: 21.3 vs. 19.9 months [HR: 0.89; *P* = 0.077]) [7]. In the second-line setting, the RR rate of various chemotherapeutic regimens has not been satisfactory, ranging from 4 % for FOLFIRI after the first-line FOLFOX6 to 15 % for FOLFOX6 after the first-line FOLFIRI and 20 % for XELOX after irinotecan-based therapies [8, 9]. Again, benefit of adding bevacizumab was demonstrated in several clinical trials in this setting. Giantonio et al. reported that bevacizumab plus FOLFOX4 showed significantly better survival data compared with FOLFOX4 alone after the first-line irinotecan-based treatment (OS 12.9 vs. 10.8 months [HR: 0.75; *P* = 0.011], PFS 7.3 vs. 4.7 months [HR: 0.61; *P* < 0.001], RR: 22.7 vs. 8.6 % [*P* < 0.001]) [10]. Bennouna et al. showed that bevacizumab plus irinotecan–based regimens showed efficacy with acceptable safety profile after the first-line oxaliplatin-based treatments (PFS: 7.8, OS: 22.4, and RR: 33 %) [11].

More recently, a survival benefit associated with the continuous use of bevacizumab beyond progression (BBP) was generated by two large studies. A large observational cohort study that evaluated the efficacy and safety of bevacizumab in combination with chemotherapy (BRiTE study) indicated that the BBP could contribute to prolong the OS [12]. The Avastin registry: investigation of effectiveness and safety (ARIES) also looked at the role of BBP and indicated trend toward longer OS among patients who received bevacizumab beyond first progression compared with patients who received bevacizumab only after progression (27.5 vs. 18.7 months) [13]. However, these are observational studies, and true benefits and risks of BBP are yet to be shown in a prospective clinical trial, particularly in Japan. This prompted us to conduct a multicenter phase II study of mFOLFOX6 plus bevacizumab followed by FOLFIRI plus bevacizumab in mCRC to explore the BBP strategy for the first time in the Japanese population.

## Patients and methods

### Patients

The study inclusion criteria were histologically confirmed colorectal adenocarcinoma; unrespectable metastatic disease; age 20 years or older; Eastern Cooperative Oncology Group (ECOG) performance status of 0 or 1; no previous chemotherapy for mCRC; bidimensionally measurable disease; a life expectancy of at least 3 months; adequate organ function (white blood cell count 3,000–12,000 cells per μL, neutrophilic cell count ≥1,500 cells per μL, platelet count ≥100,000 per μL, aspartate aminotransferase [AST] ≤100 IU/L, alanine aminotransferase [ALT] ≤100 IU/L, total bilirubin ≤25.7 μmol/L [≤15 mg/L], and creatinine ≤106.1 μmol/L [≤12 mg/L]). Exclusion criteria were pregnancy or lactation; second non-colorectal cancer; complications such as ileus, uncontrolled diabetes mellitus, or hypertension; severe diarrhea; clinically evident gastrointestinal hemorrhage; and ascites or pleural effusion needing treatment. The protocol of this study was approved by the institutional review board or ethics committee of each institution. The study was conducted in compliance with the Declaration of Helsinki. Written informed consent was obtained from all patients participating in the study.

### Treatment plan

As the first-line setting for mCRC, the patients received bevacizumab plus mFOLFOX6 therapy (consisting of bevacizumab [5 mg/kg], oxaliplatin [85 mg/m^2^], and folinic acid [200 mg/m^2^] followed by bolus infusion of fluorouracil [400 mg/m^2^] and subsequent continuous infusion of fluorouracil [2,400 mg/m^2^], repeated every 2 weeks) until disease progression, unacceptable toxicity, or patient’s wish to terminate the treatment. In the subsequent second-line setting, the patients received bevacizumab plus FOLFIRI therapy (consisting of bevacizumab [5 mg/kg], irinotecan [150 mg/m^2^], and folinic acid [200 mg/m^2^] followed by bolus infusion of fluorouracil [400 mg/m^2^] and subsequent continuous infusion of fluorouracil [2,400 mg/m^2^], repeated every 2 weeks) until disease progression, unacceptable toxicity, or patient’s wish to terminate the treatment.

Surgical treatment of the metastatic lesions was allowed in patients with sufficient objective response that rendered the lesions resectable.

### Assessments

**Fig. 1:**
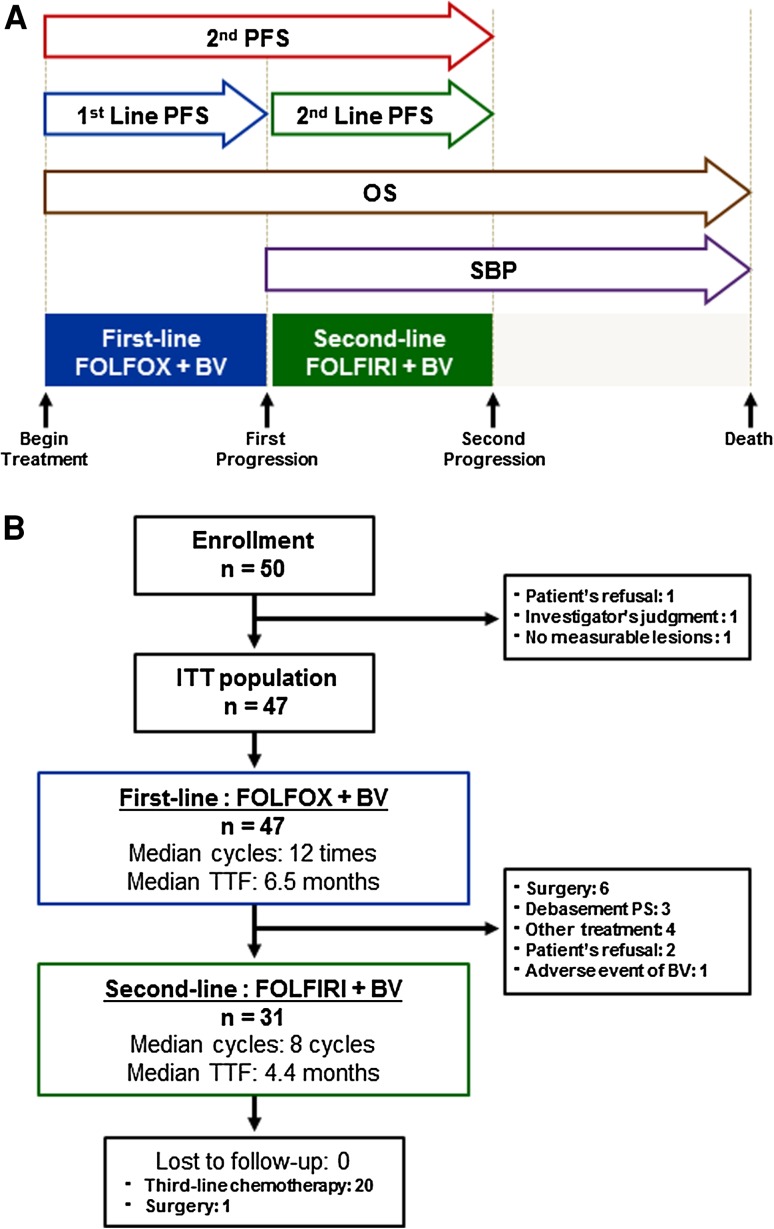
Schematic of patient observation periods (**a**) and consort chart of the patients (**b**). **a** The second progression-free survival (2nd PFS) is measured from the start of first-line treatment to disease progression after second-line treatment. Progression-free survival (PFS) of each therapy is measured from the start of each therapy to disease progression. Survival beyond first progression (SBP) is measured from the first progression to death. Overall survival (OS) is measured from the start of first-line treatment to death. **b** Fifty patients were enrolled in this study. Three patients were excluded from the study. Forty-seven patients who received the protocol treatment were included in the safety evaluation

The primary objective of this study was the second progression-free survival (2nd PFS), defined as the time duration from the date of initiation of the first-line therapy until investigator-assessed disease progression or patient death due to any cause after starting the second-line treatment. If the patient could not receive second-line treatment for medical reasons or refusal, progression-free survival (PFS) on first-line therapy was used. Secondary objectives were OS (the time duration from the date of initiation of each therapy to death due to any cause), survival beyond first progression (SBP) (the time duration from the date of first disease progression to death due to any cause), PFS (the time duration from the date of initiation of each therapy to disease progression or death due to any cause), RR (the proportion of patients who achieved a best response of either a complete response [CR] or partial response [PR] during each therapy), disease control rate (DCR) (the proportion of patients with CR, PR, or stable disease [SD] during each therapy), and safety. Schematic of patients observation periods is presented in Fig. 1b. Adverse events were assessed using National Cancer Institute Common Toxicity Criteria (NCI-CTC), version 3.0. In addition, the frequency of bevacizumab-related adverse events (gastrointestinal perforation, wound healing complications, bleeding, hypertension, proteinuria, and thromboembolic events) was assessed.

### Statistical analysis

Assuming a threshold for 2nd PFS of 10.5 months and an expected 2nd PFS of 15.8 months, referring to data from the previous clinical trials, and a 2-year enrollment period and a 2-year follow-up period, 44 patients in total were required to ensure an alpha error of 0.05 (one-sided) and detection power (1-β) of 80 %. Taking possible dropouts into consideration, the sample size of this study was determined as 50. The 2nd PFS, the primary objective of this study, was estimated using the Kaplan–Meier method, and the median 2nd PFS and its 95 % confidence interval were estimated. Other time-to-endpoint data, PFS and OS, were also estimated in the same manner. RR, DCR, and the toxicities were calculated as proportions with exact confidence intervals.

## Results

### Patient characteristics

Fifty patients from 12 institutions in Japan were enrolled in this study from August 2008 to May 2010. Three patients were excluded from the study: one due to the patient’s refusal, one due to the investigator’s decision, and one due to no measurable lesions as per the inclusion criteria. Forty-seven patients who received the protocol treatment were included in the evaluation of efficacy and safety. Baseline characteristics and consort chart of the patients are presented in Table 1 and Fig. 1b.

**Table 1:** Patient characteristics

Parameter	No. of patients (*N* = 47)	%
Age, years		
Median	63 40–74	
Range		
Sex		
Male	31	66.0
Female	16	34.0
Performance status WHO		
0	29	61.7
1	18	38.3
Primary site		
Colon	31	66.0
Rectum	16	34.0
Metastases		
Synchronous	7	14.9
Metachronous	40	85.1
Metastatic sites		
Liver	21	44.6
Lung	21	44.6
Peritoneum	1	2.1
Lymph nodes	10	21.3
Adjuvant chemotherapy		
No	27	57.4
Yes	20	42.6
5FU-based	20	42.6
Oxaliplatin-based	0	0

*No/N* number, *WHO* World Health Organization, *5FU* 5-fluorouracil

### Treatment status

As the first-line treatment, 47 patients received a median of 12 cycles (range 2–39) of bevacizumab plus mFOLFOX6 therapy. Median time-to-treatment failure (TTF) was 6.5 months (95 % CI, 4.0–9.0 months). The median relative dose intensity (RDI) for bevacizumab and oxaliplatin was 88 and 76 %. Treatment was discontinued because of disease progression in 21 patients (44.7 %), adverse events in 14 patients (29.8 %), and patient’s refusal in two patients (4.3 %). Secondary surgery to remove metastases was performed in six patients (12.8 %).

As for the second-line treatment, 31 patients received a median of eight cycles (range, 2–28) of bevacizumab plus FOLFIRI therapy. Median TTF was 4.4 months (95 % CI, 2.4–6.4 months). The median RDI for bevacizumab and irinotecan was 80 and 76 %. Treatment was discontinued because of disease progression in 20 patients (64.5 %) and adverse events in two patients (6.5 %). Secondary surgery to remove metastases was performed in one patient (3.2 %). After undergoing the second-line protocol treatment, 20 patients (64.5 %) received a third-line chemotherapy, of which the regimen delivered to six patients (19.4 %) was cetuximab.

There was no therapy-related death in this study. Treatment status is summarized in Table 2.

**Table 2:** Treatment status

	First-line therapy (mFOLFOX6 + BV) (*N* = 47)	Second-line therapy (FOLFIRI + BV) (*N* = 31)
Treatment cycle (times)		
Median	12	7
Range	2–39	2–26
Time-to-treatment failure (month)		
Median	6.5	3.8
95 % CI	4.0–9.0	2.7–4.5
Median relative dose intensity (%)		
Bevacizumab	88	80
Oxaliplatin	76	–
Irinotecan	–	76
Reasons for discontinuation (%)		
Progression of disease	44.7	64.5
Toxicity	29.8	6.5
Secondary surgery for metastasis (%)	12.8	3.2

*BV* bevacizumab, *N* number, *CI* confidence interval

### Clinical outcomes

After a median follow-up period of 35.9 months (range, 24.2–44.8 months), 39 disease progressions (83.0 %) and 26 deaths (55.3 %) occurred in the 47 patients enrolled.

**Fig. 2:**
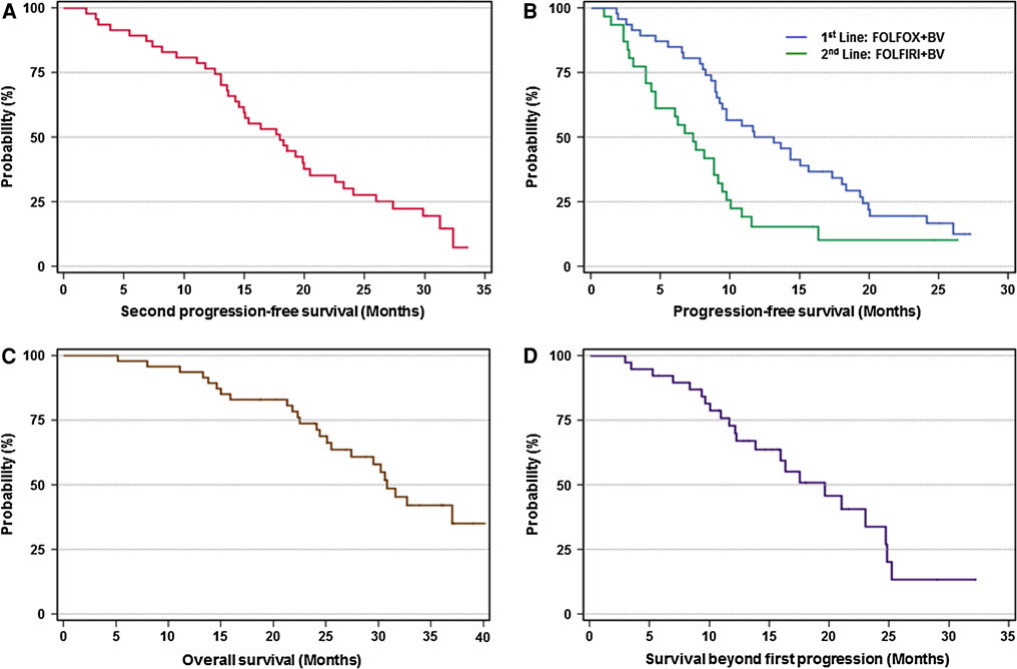
Survival outcomes. **a** Median second progression-free survival, the primary endpoint, was 17.7 months (95 % CI, 13.4–22.0 months). **b** Median progression-free survivals were 13.1 months (95 % CI, 8.7–17.5 months) in the first-line setting and 7.5 months (95 % CI, 4.9–10.2 months) in the second-line setting. **c** Median overall survival was 30.6 months (95 % CI, 13.4–22.0 months). **d** Median survival beyond the first progression was 17.7 months (95 % CI, 13.4–22.0 months). Survival curves were estimated using Kaplan–Meier methods

Median 2nd PFS, the primary endpoint, was 18.0 months (95 % CI, 13.7–22.3 months) (Fig. 2a).

Median OS was 30.8 months (95 % CI, 27.7–34.0 months) (Fig. 2c), and median SBP was 19.6 months (95 % CI, 13.5–25.7 months) (Fig. 2d).

In the first-line bevacizumab plus mFOLFOX6 therapy, RR and DCR of the 47 patients were 61.7 and 89.4 %, respectively (five patients had CR, 24 patients had PR and 13 patients had SD) (Table 3). The median PFS from the initiation of the first-line therapy was 13.1 months (95 % CI, 8.7–17.5 months) (Fig. 2b).

**Table 3:** Objective tumor response

Response	First-line therapy (*N* = 47)		Second-line therapy (*N* = 31)	
	No. of patients	%	No. of patients	%
CR	5	10.6	2	6.5
PR	24	51.1	7	22.6
SD	13	27.7	11	35.5
PD	5	10.6	11	35.5
RR (%)	61.7 89.4		29.0 64.5	
DCR (%)				

*No/N* number, *CR* complete response, *PR* partial response, *SD* stable disease, *PD* progressive disease, *RR* response rate (CR + PR), *DCR* disease control rate (CR + PR + SD)

In the second-line bevacizumab plus FOLFIRI therapy, RR and DCR of the 31 patients who went on to the second-line therapy were 29.0 and 64.5 %, respectively (two patients had CR, seven patients had PR, and 11 patients had SD) (Table 3). The median PFS from the initiation of the second-line therapy was 7.3 months (95 % CI, 5.0–9.6 months) (Fig. 2b).

### Adverse events

Frequency of common toxicities is presented in Table 4. The incidences of hematologic and non-hematologic > grade 3 toxic events were 44.4 and 16.7 %. The hematologic toxic events (>grade 3) occurred in 10 patients (50 %) in the first-line therapy and six patients (37.5 %) in the second-line therapy. The non-hematologic toxic events (>grade 3) occurred in five patients (25 %) in the first-line therapy and no patient (0 %) in the second-line therapy.

**Table 4:** Frequency of common toxicities

Toxicity	First-line therapy (*N* = 47)		Second-line therapy (*N* = 31)	
	All grades (%)	>Grade 3 (%)	All grades (%)	>Grade 3 (%)
Hematologic toxicity	72.3	27.7	51.6	32.3
Neutropenia	57.4	23.4	41.9	22.6
Thrombocytopenia	12.8	0	9.7	0
Anemia	23.4	0	9.7	0
Febrile neutropenia	–	4.3	–	3.2
Non-hematologic toxicity	85.1	25.5	51.6	12.9
Diarrhea	0	0	12.9	3.2
Nausea/vomiting	27.7	4.3	19.4	0
Mucositis	10.6	2.1	12.9	3.2
Hand-foot syndrome	2.1	0	0	0
Alopecia	2.1	0	3.2	0
Fatigue	6.4	0	3.2	3.2
Neuropathy	72.3	17.0	19.4	3.2
Allergy	12.8	2.1	3.2	0
Bevacizumab-associated toxicity	51.1	2.1	45.2	3.2
Hypertension	25.5	0	45.2	3.2
Proteinuria	21.3	0	16.1	0
Bleeding	2.1	0	3.2	0
Infection	2.1	0	0	0
Thrombosis	2.1	0	0	0
GI perforation	2.1	2.1	0	0

*N* number, *GI* gastrointestinal

Severe adverse events associated with bevacizumab during the first-line therapy were grade 3 GI perforation in one case (2 %), grade 2 venous thromboembolic event in one case (2 %), and grade 2 bleeding event in one case (2 %). However, no critical events associated with bevacizumab were observed during the second-line therapy. There was a higher incidence of new or worsening hypertension in the second-line therapy as compared with the first-line therapy (26 vs. 45 %).

## Discussion

This is the first prospective study to examine the continuous use of bevacizumab in combination with FOLFIRI after failing the first-line treatment with mFOLFOX/bevacizumab combination in the Japanese patients with mCRC. There are several issues regarding the use of BBP that needs to be clarified; the response and survival benefit obtained through adding bevacizumab to each line of chemotherapy, the survival benefit of the BBP strategy *per se*, and the adverse effect of long-term exposure to bevacizumab among patients who received BBP. Of these, benefits in terms of response rate and survival by adding bevacizumab to either the first-line oxaliplatin-based chemotherapy or second-line irinotecan-based chemotherapy have been well documented in previous clinical trials [7, 11]. In the current study, the response and survival data observed both in the first-line and second-line settings seem to compare favorably with these studies, with a RR of 61.7 % and a PFS of 13.1 months in the first-line setting, and a RR of 29.0 % and a PFS of 7.5 months in the second-line setting.

In general, failure to respond to chemotherapy with cytotoxic agents implies inherent or acquired resistance to the therapy and leads to a change in the therapeutic regimen. The mechanisms of the resistance to cytotoxic agents are typically consequences of genetic instability inherent in cancer that renders mutant cells insensitive to chemotherapeutic agents. In contrast, the mechanisms of resistance to biologic targeted agents, including bevacizumab, are not well understood. One hypothesis that forms the basis of BBP is that persistent VEGF suppression continues to have clinical benefit when given in combination with the secondary and tertiary cytotoxic regimens. This hypothesis was supported by the results of several clinical trials exploring benefit of BBP. The first evidence of a survival benefit associated with BBP was generated by a large, observational study, BRiTE study. In this study, the patients who had been treated with BBP had a superior median SBP and OS (19.2 and 31.8 months, respectively) as compared with those who were treated without BBP (9.5 and 19.9 months, respectively) [12]. The ARIES study examined the role of bevacizumab after disease progression in patients who had received first-line bevacizumab and in those who were bevacizumab-naive at the time of second-line treatment. The authors observed a trend toward longer SBP and OS in patients who had received first-line and second-line bevacizumab (median SBP: 14.1 and OS: 27.5 months) when compared with patients who received bevacizumab only after the disease progression (median SBP: 7.5 and OS: 18.7 months), while PFS of the second-line treatment was similar in both groups [13].

The primary objective of the current study was to assess the efficacy of BBP determined in terms of the 2nd PFS, defined as the time duration from the initiation of the first-line therapy until disease progression during the second-line of chemotherapy. Tournigand et al. reported that the median 2nd PFS was 10.9 months when the first-line FOLFOX and second-line FOLFIRI were administered, both without bevacizumab, and this was a historical benchmark to design our study. The median 2nd PFS of 17.7 months as shown in this study met our expectations and clearly pointed to an improvement in the outcome compared with the historical precedent setting without bevacizumab. There could be an argument that the endpoint of a chemotherapeutic strategy such as BBP that constitutes from several lines of treatment should be OS. In this aspect, the median OS and SBP in this study were 30.8 and 19.6 months, respectively. These survival data are potentially comparable with the results observed in the BBP population from the previous studies.

Safety of a long-term exposure to bevacizumab among patients who received BBP is another issue explored in this study. The safety outcomes in the BRiTE study showed no apparent increase in serious adverse events reported in the BBP group compared with the no-BBP group [12, 14], with the exception of thromboembolic event in the elderly population [15]. Such thromboembolic event was rare at 2 % in the current Japanese population. Other severe adverse events associated with bevacizumab were grade 2 bleeding event (2 %) and grade 3 GI perforation (2 %), all of which occurred during the first-line chemotherapy. Thus, no critical events associated with bevacizumab were observed during the second-line therapy. It is of note that a higher incidence of new or worsening hypertension was observed during the second-line therapy compared with the first-line therapy. The higher cumulative incidence of hypertension in the BBP group was not unexpected, given that the risk of developing bevacizumab-associated hypertension appears to accumulate over time and that the BBP results in substantially longer bevacizumab exposure. The type and frequency of other grade 3/4 events (including neutropenia, diarrhea, vomiting, and asthenia) were consistent with the known safety profile of the chemotherapy regimens.

Our study is merely hypothesis-generating regarding the efficacy of BBP because of the one-arm design and relatively small sample size. However, it does imply that the BBP strategy is beneficial to the Japanese population with the 2nd PFS nearly 10 months longer than that observed in the Tournigand study and SBP and OS that is similar to the survival data observed in the BRiTE study and the ARIES study. Data regarding safety of the BBP strategy was more robust, in which only hypertension was to be carefully taken care of. From these encouraging data, it can now be recommended that a randomized study involving a larger numbers of patients be performed in Japan to obtain hard evidence regarding the efficacy of BBP.

In summary, the planned continuation of bevacizumab during the second-line treatment is feasible for the Japanese mCRC patients. A prospective randomized control study to confirm the efficacy is warranted.
